# Impact of renal dysfunction on long-term outcomes of elderly patients with acute coronary syndrome: a longitudinal, prospective observational study

**DOI:** 10.1186/1471-2369-15-78

**Published:** 2014-05-09

**Authors:** Yuqi Liu, Lei Gao, Qiao Xue, Muyang Yan, Pu Chen, Yu Wang, Yang Li

**Affiliations:** 1 Institute of Geriatric Cardiology, Chinese PLA General Hospital, Beijing 100853, China; 2 Kidney Department, Chinese PLA General Hospital, Beijing 100853, China

**Keywords:** Acute coronary syndrome, Glomerular filtration rate, Renal dysfunction, MACE, Elderly

## Abstract

**Background:**

This study investigated the impact of renal dysfunction (RD) on long-term outcomes in elderly patients with acute coronary syndrome (ACS), and evaluated prognostic factors in elderly patients with ACS and RD.

**Methods:**

This longitudinal prospective study included 184 consecutive patients who were admitted with ACS between January 2009 and January 2010 and also had RD. Patients were divided into five groups according to their estimated glomerular filtration rate (eGFR): 1) eGFR ≥ 90 mL/min/1.73 m^2^ with evidence of kidney damage, 2) 60 ≤ eGFR < 90 mL/min/1.73 m^2^, 3) 30 ≤ eGFR < 60 mL/min/1.73 m^2^, 4) 15 ≤ eGFR < 30 mL/min/1.73 m^2^, and 5) eGFR < 15 mL/min/1.73 m^2^. The primary endpoints were death and complications during hospitalization. The secondary endpoint was any major adverse cardiac event (MACE) during follow-up.

**Results:**

The mean follow-up period was 502.2 ± 203.6 days. The mean patient age was 73.7 ± 9.4 years, and 61.4% of the patients were men. Severe RD (eGFR < 30 mL/min/1.73 m^2^) was an independent predictor of MACE. Severe RD was associated with a low hemoglobin level, low left ventricular ejection fraction, and high levels of high-sensitivity C-reactive protein, N-terminal pro-B-type natriuretic peptide, and cystatin C. Survival was significantly poorer in patients with severe RD than in patients with mild RD.

**Conclusions:**

Among patients with ACS, severe RD was associated with advanced age, diabetes, hypertension, and cardiac dysfunction. Severe RD was an independent risk factor for MACE, and was associated with poor prognosis.

## Background

Morbidity associated with chronic kidney disease (CKD) has increased with the growth of aging populations. CKD is strongly associated with increased mortality rate and accelerated cardiovascular disease (CVD) [1]. Patients with CKD have complex clinical conditions and a poor prognosis, and are difficult to treat [2-4]. One of the main concerns for patients with CKD is the increased risk of CVD, including coronary heart disease, cerebrovascular disease, and peripheral vascular disease [5-10]. A meta-analysis found that individuals with renal insufficiency have an approximately 3-fold increased risk of CVD mortality compared with their counterparts without renal insufficiency [5]. Despite strong evidence linking CKD to poor outcomes, the impact of CKD on mortality and morbidity in elderly patients with acute coronary syndrome (ACS) is probably underappreciated, and elderly patients with CKD may not be treated as aggressively as patients with normal renal function. This study evaluated the impact of renal dysfunction (RD) on clinical outcomes including death, complications, and major adverse cardiac events (MACEs) in elderly patients with ACS.

## Methods

### Study population

We analyzed the data of 184 consecutive elderly patients (all older than 60 years, mean age 73.7 ± 9.4 years, 61.4% men) who were admitted to our department with ACS between January 2009 and January 2010. All patients were discharged with a diagnosis of ACS based on cardiac enzyme levels and electrocardiography findings. Coronary angiography was performed in 129 patients. The secondary endpoint was analyzed in 147 patients, excluding 18 patients who were transferred to other hospitals or died, and 19 patients who were lost to follow-up. The ethics committee of the Chinese PLA General Hospital approved this study, and written informed consent for inclusion in the study was obtained from all subjects.

### Definitions

The abbreviated Modification of Diet in Renal Disease formula was used to calculate the estimated glomerular filtration rate (eGFR) from the serum creatinine level [11]. Patients were divided into five groups according to their eGFR: Group I, eGFR ≥ 90 mL/min/1.73 m^2^ (n = 13, age 63.3 ± 5.1 years, 61.5% men); Group II, 60 ≤ eGFR < 90 mL/min/1.73 m^2^ (n = 15, age 65.2 ± 7.4 years, 73.3% men); Group III, 30 ≤ eGFR < 60 mL/min/1.73 m^2^ (n = 81, age 76.0 ± 7.6 years, 69.2% men); Group IV, 15 ≤ eGFR < 30 mL/min/1.73 m^2^ (n = 45, age 74.3 ± 8.7 years, 53.3% men); and Group V, eGFR ≤ 15 mL/min/1.73 m^2^ (n = 30, age 75.2 ± 12.9 years, 60% men).

Acute kidney injury is defined as a rapid reduction in renal function characterized by progressive azotemia (determined by the serum creatinine level), with or without oliguria. Acute kidney injury is categorized as Stage 1 if there is an increase in the serum creatinine level of 50% or ≥ 0.3 mg/dL within 48 h, Stage 2 if there is an increase in the serum creatinine level of ≥ 100% (doubling), or Stage 3 if there is an increase in the serum creatinine level of ≥ 200% or of 0.5 mg/dL to at least 4.0 mg/dL.

The primary endpoints were death and complications during hospitalization. The secondary endpoint was any MACE during the follow-up period, including cardiac death, myocardial infarction, stroke, and emergency or elective repeat revascularization. Cardiac death was defined as mortality not resulting from noncardiac disease.

The infarct-related artery was defined according to the American College of Cardiology/American Heart Association classification and the Thrombolysis In Myocardial Infarction flow grade [12]. Target lesion revascularization was defined as repeat revascularization with stenosis of ≥ 50% in the treated lesion. Target vessel revascularization was defined as repeat revascularization of the treated vessel. MACE was defined as any of cardiac death, myocardial infarction, target lesion revascularization, or target vessel revascularization. Other major bleeding was defined as severe bleeding other than intracranial bleeding. Death and complications during hospitalization were recorded, including cardiogenic shock, ventricular tachycardia or fibrillation requiring anti-arrhythmic drugs or defibrillation, atrioventricular block requiring temporary cardiac pacemaker insertion, recurrent myocardial ischemia or infarction, stroke, major bleeding, and acute kidney injury. If more than two complications occurred in a single patient, each complication type was recorded. After discharge, any MACEs during the follow-up period were recorded.

### Clinical data collection

Laboratory data were collected on admission, including the levels of total cholesterol, low-density lipoprotein, hemoglobin, high-sensitivity C-reactive protein (hs-CRP), N-terminal pro-B-type natriuretic peptide (NT-proBNP), serum creatinine, troponin-T, creatine kinase-MB, and cystatin C (Cys C). Echocardiographic parameters were assessed by transthoracic echocardiography using the Teichholz method before coronary angiography, including thickness of the interventricular septum, left ventricular end-diastolic inner volume, left ventricular posterior wall thickness, and left ventricular ejection fraction.

### Statistical analysis

Statistical analyses were performed using the Statistical Package for Social Sciences software (SPSS version 13.0). Continuous variables with normal distributions were expressed as mean ± standard deviation and compared using 1-way analysis of variance. Categorical variables were compared using the chi-square test where appropriate. MACE was estimated by the unadjusted Kaplan–Meier method in the five eGFR groups. Cox proportional hazards modeling was used to examine the relationships between survival and a prespecified list of risk factors for MACE, including kidney function. The analyses were adjusted for hypertension, diabetes mellitus, smoking, sex, hyperlipidemia, age, hemoglobin level, surgical interventions, and medications (including aspirin, beta-blockers, angiotensin-converting enzyme inhibitors, calcium channel blockers, and statins).

## Results

### Baseline characteristics

A total of 184 patients were included in this study, including 13 (7.1%) with ST-segment elevation myocardial infarction, 48 (26.1%) with non-ST-segment elevation myocardial infarction, and 123 (66.8%) with unstable angina. The median follow-up period was 502.2 ± 203.6 days. The mean patient age was 73.7 ± 9.4 years, and 61.4% of the patients were men. Patients with severe RD (eGFR < 30 mL/min/1.73 m^2^) had higher rates of comorbid conditions than those without severe RD, including hypertension (n = 48, 64.0%) diabetes mellitus (n = 48, 64.0%), current smoking (n = 25, 33.3%), hyperlipidemia (n = 15, 20.0%), and a history of ischemic heart disease (n = 44, 58.6%). More severe RD was significantly associated with older age, hypertension, hyperlipidemia, ischemic heart disease, and high Killip class (p < 0.05) (Table 1).

**Table 1:** Baseline characteristics

	**Group I N = 13**	**Group II N = 15**	**Group III N = 81**	**Group IV N = 45**	**Group V N = 30**	***P***
Age (yrs)	63.3 ± 5.1	65.2 ± 7.4	76.0 ± 7.6	74.2 ± 8.7	75.2 ± 12.9	0.000
Male (%)	8 (61.5)	11 (73.3)	48 (69.2)	24 (53.3)	18 (60.0)	NS
BMI (kg/m^2^)	25.9 ± 1.7	27.3 ± 5.2	25.6 ± 1.6	23.8 ± 2.9	23.9 ± 2.1	0.000
Risk factor (%)						
Hypertension	3 (23.1)	12 (80.0)	51 (62.9)	21 (46.7)	27 (90.0)	0.001
DM	8 (61.5)	9 (60.0)	27 (33.3)	15 (33.3)	15 (50.0)	NS
Smoking	3 (23.1)	5 (33.3)	19 (23.5)	17 (37.7)	8 (26.7)	NS
Hyperlipidemia	8 (61.5)	9 (60.0)	18 (22.2)	6 (13.3)	9 (30.0)	0.0003
IHD	3 (23.1)	7 (46.7)	30 (37.0)	22 (48.9)	22 (73.3)	0.005
Symptom at admission (%)						
Chest pain	8 (61.5)	10 (66/7)	39 (50.0)	29 (64.4)	21 (70.0)	NS
Dyspnea	0 (0.0)	1 (6.7)	5 (6.4)	3 (6.7)	2 (6.7)	NS
Killip class	1.67 ± 1.1	1.67 ± 1.2	2.3 ± 1.2	2.5 ± 1.1	3.3 ± 0.5	0.000
Diagnosis						NS
UA	8 (61.5)	11 (73.3)	54 (6.7)	27 (60.0)	21 (70.0)	NS
NSTEMI	5 (38.5)	2 (13.3)	23 (28.4)	15 (33.3)	6 (20.0)	NS
STEMI	0 (0)	2 (13.3)	4 (4.9)	3 (6.7)	3 (10.0)	NS

DM, diabetes mellitus; IHD, ischemic heart disease; STEMI, ST-segment elevation myocardial infarction; NSTEMI, non-ST-segment myocardial infarction; UA, unstable angina; NS, not significant.

### Biochemical parameters and echocardiography findings

Table 2 shows the baseline continuous variables. More severe RD was associated with a lower hemoglobin level, lower left ventricular ejection fraction, and higher levels of hs-CRP, NT-proBNP, and Cys C. The thickness of the interventricular septum, left ventricular end-diastolic inner volume, and left ventricular posterior wall thickness were greater in patients with severe RD than in patients with mild RD.

**Table 2:** Biochemical parameters and echocardiogram findings

	**Group I N = 13**	**Group II N = 15**	**Group III N = 81**	**Group IV N = 45**	**Group V N = 30**	***P***
GFR (mL/min)	106.8 ± 9.2	69.2 ± 6.1	42.9 ± 9.4	24.7 ± 7.5	10.9 ± 3.4	0.013
Creatinine (mg/dL)	64.1 ± 20.1	103.0 ± 19.4	126.6 ± 40.5	331.4 ± 148.4	497.3 ± 377.9	0.003
Troponin T (ng/mL)	0.12 ± 0.10	0.09 ± 0.04	0.41 ± 0.33	0.21 ± 0.09	0.59 ± 0.49	0.918
CK-MB (U/L)	7.3 ± 2.9	9.9 ± 3.6	13.1 ± 4.6	13.2 ± 3.7	17.2 ± 9.7	0.627
Hgb (g/dL)	123.1 ± 12.1	135.8 ± 13.9	127.6 ± 18.2	107.1 ± 23.7	93.2 ± 22.9	0.000
TC (mmol/L)	3.6 ± 1.2	5.3 ± 1.6	4.3 ± 1.1	4.4 ± 1.6	3.4 ± 0.9	0.159
LDL-C (mmol/L)	2.3 ± 0.9	2.9 ± 1.2	2.4 ± 0.7	2.5 ± 1.3	1.8 ± 0.6	0.257
hs-CRP (mg/dL)	0.6 ± 0.5	1.1 ± 0.7	1.7 ± 0.9	2.7 ± 1.2	4.9 ± 3.7	0.000
Cys C (mg/l)	0.6 ± 0.3	1.0 ± 0.4	1.4 ± 0.5	2.4 ± 1.2	3.7 ± 2.8	0.000
NT-proBNP (pg/mL)	307.6 ± 52.4	1013.8 ± 639.5	4305.5 ± 1054.5	9687.3 ± 3014.7	10636.8 ± 4016.9	0.000
LVEF (%)	62 ± 13	53 ± 13	48 ± 11	49 ± 10	46 ± 12	0.007
IVS (mm)	9.3 ± 1.5	10.8 ± 1.8	11.2 ± 1.3	11.9 ± 1.4	12.2 ± 1.8	0.021
LVPWT (mm)	9.3 ± 1.4	11.4 ± 2.3	10.6 ± 1.2	11.2 ± 1.7	12.0 ± 1.1	0.028
EDV (ml)	48.0 ± 3.0	48.8 ± 6.2	49.4 ± 5.9	50.5 ± 6.8	56.2 ± 5.6	0.046

GFR, glomerular filtration rate; Hgb, hemoglobin; TC, total cholesterol; LDL, low-density lipoprotein; hs-CRP, high-sensitivity C-reactive protein; NT-proBNP, N-terminal pro-B-type natriuretic peptide; CK-MB, creatine kinase-MB; NS, not significant; Cys C, cystatin C; LVEF, left ventricular ejection fraction; IVS, interventricular septal thickness; LVPWT, left ventricular posterior wall thickness; EDV, left ventricular end-diastolic inner volume.

### Coronary angiography results

Table 3 shows the coronary angiography data with different groups eGFR groups. A total of 129 (70.1%) patients underwent coronary angiography. The number of involved vessels did not differ among the groups; patients with severe RD had more complex lesions in the left main coronary artery and in the infarct-related artery compared with the mild renal dysfunction group.

**Table 3:** Baseline coronary angiographic findings

	**Group I N = 13**	**Group II N = 15**	**Group III N = 81**	**Group IV N = 45**	**Group V N = 30**	***P***
CAG (%)	13 (100)	15 (100)	63 (77.8)	23 (51.1)	15 (50)	0.000
Infarct-related artery (%)						
LAD	8 (61.5)	10 (66.7)	41 (65.1)	11 (47.8)	7 (46.7)	0.001
LCx	1 (7.7)	1 (6.7)	7 (11.1)	3 (13.0)	1 (6.7)	NS
RCA	4 (30.8)	3 (20.0)	13 (20.6)	7 (30.4)	4 (26.7)	NS
LM	0 (0)	1 (6.7)	2 (3.2)	2 (8.7)	3 (20.0)	NS
Involved vessel number (%)						
1 Branch	7 (53.8)	8 (53.3)	23 (36.5)	11 (47.8)	5 (33.3)	NS
2 branch	4 (30.8)	4 (26.7)	32 (50.8)	7 (30.4)	3 (20.0)	NS
3 branch	2 (15.4)	2 (13.3)	7 (11.1)	2 (8.7)	4 (26.7)	NS
LM, isolated	0 (0)	1 (6.7)	1 (1.6)	1 (4.3)	1 (6.7)	NS
LM, complex	0 (0)	0 (0)	0 (0)	2 (8.7)	2 (13.3)	0.032
ACC / AHA classification						
A	7 (53.8)	5 (33.3)	21 (33.3)	2 (8.7)	3 (20.0)	0.045
B1	4 (30.8)	6 (40.0)	22 (34.9)	8 (34.8)	2 (13.3)	NS
B2	2 (15.4)	3 (20.0)	13 (20.6)	7 (30.4)	4 (26.7)	NS
C	0 (0)	1 (6.7)	7 (11.1)	6 (26.1)	6 (40.0)	0.011
TIMI flow (%)						
TIMI 0	0 (0)	3 (20.0)	4 (6.3)	4 (17.4)	3 (20.0)	NS
TIMI 1	1 (7.7)	1 (6.7)	2 (3.2)	2 (8.7)	1 (6.7)	NS
TIMI 2	3 (23.1)	1 (6.7)	13 (20.6)	2 (8.7)	1 (6.7)	NS
TIMI 3	9 (69.2)	10 (66.7)	44 (69.8)	15 (65.2)	10 (66.7)	NS

LM, left main; ACC/AHA, American College of Cardiology/American Heart Association; TIMI, thrombolysis in myocardial infarction; NS, not significant.

### Risk factors and outcomes

**Figure 1:**
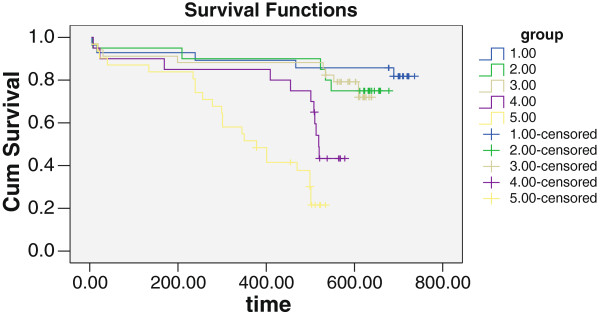
Kaplan-Meier survival in elderly patients with ACS.

Table 4 shows the in-hospital and out-of-hospital outcomes according to eGFR groups. In patients with normal renal function, the estimated in-hospital death rate was 0% and the estimated in-hospital complication rate was 7.7%. Major bleeding occurred in one patient, who was in Group I. Cardiogenic shock occurred in five patients. Atrioventricular block occurred in four patients and ventricular tachycardia occurred in four patients, who were all in Group V. In patients with moderate RD, the in-hospital death rate (3.9%) and the in-hospital complication rate (20.6%) were significantly higher than in Group I (both p < 0.05). The incidence of MACE during follow-up was higher in Group V than in Group I (p < 0.05). Figure 1 shows the Kaplan–Meier analyses for MACE-free survival during follow-up according to eGFR groups. Patients with severe RD had a lower probability of event-free survival than patients in the other groups. Cox multivariate regression analysis showed that eGFR was the main independent predictor of MACE (odds ratio 0.953, 95% confidence interval 0.926–0.982, p = 0.0004).

**Table 4:** Outcomes according to estimated glomerular filtration rate

	**Group I N = 13**	**Group II N = 15**	**Group III N = 81**	**Group IV N = 45**	**Group V N = 30**	***P***
In-hospital outcome						
Death (%)	0	1 (6.7)	4 (4.9)	2 (4.4)	6 (20)	0.046
Complications (%)	1 (7.7)	1 (6.7)	5 (6.7)	4 (8.8)	8 (26.7)	0.031
AKI	0	1 (6.7)	2 (2.5)	1 (2.2)	2 (6.7)	NS
Cardiogenic shock	0	1 (6.7)	2 (2.5)	4 (8.8)	5 (16.7)	NS
Major bleeding	1 (7.7)	0	1 (1.2)	1 (2.2)	1 (3.3)	NS
AV block	0	0	1 (1.2)	2 (4.4)	4 (13.3)	0.039
VT	0	0	1 (1.2)	2 (4.4)	4 (13.3)	0.039
Hospital stay (days)	10.3 ± 10.9	19.8 ± 11.9	19.9 ± 18.8	24.5 ± 28.1	29.1 ± 12.5	0.042
Out-hospital outcome						
MACE (%)	1 (7.7)	2 (13.3)	7 (8.6)	5 (11.1)	10 (33.3)	0.015
Cardiac death	0	0	1 (1.2)	2 (4.4)	2 (6.7)	NS
MI	0	1 (6.7)	1 (1.2)	2 (4.4)	5 (16.7)	0.016
Re-PCI	1 (7.7)	2 (26.7)	3 (3.7)	4 (8.9)	4 (13.3)	NS
TVR	1 (7.7)	2 (13.3)	1 (1.2)	2 (4.4)	2 (6.7)	NS
Non-TVR	0	1 (6.7)	1 (1.2)	1 (2.2)	1 (3.3)	NS
TLR	0	1 (6.7)	1 (1.2)	1 (2.2)	1 (3.3)	NS
CABG	0	1 (6.7)	0 ()	1 (2.2)	1 (3.3)	NS
Stroke	1 (7.7)	0	0	1 (2.2)	2 (6.7)	NS

AKI, acute kidney injury; VT, ventricular tachycardia; MACE, major adverse cardiac event; PCI, percutaneous coronary intervention; TVR, target vessel revascularization; TLR, target lesion revascularization; MI, myocardial infarction; CABG, coronary artery bypass graft; NS, not significant.

Cox regression analysis of risk factors including eGFR showed that severe RD was an independent risk factor for MACE in patients with ACS, and was associated with poor prognosis. Anemia and statin drugs were also significantly associated with prognosis (p < 0.05, Table 5).

**Table 5:** COX regression analysis on different renal insufficiency and other risk factors

**Factors**	**Hazard ratio (95% CI)**	***P***
Mild RD (eGFR≧90 mL/min/1.73 m^2^)	1.000	
Moderate RD (30≦eGFR < 90 mL/min/1.73 m^2^)	0.892 (0.506 ~ 1.781)	0.393
Severe RD (15≦eGFR < 30 mL/min/1.73 m^2^)	1.983 (1.014 ~ 5.543)	0.034
Renal failure (eGFR < 15 mL/min/1.73 m^2^)	2.724 (1.051 ~ 6.955)	0.021
Hypertension	1.218 (0.371 ~ 3.995)	0.745
DM	1.971 (0.622 ~ 6.241)	0.249
Smoke	1.000 (0.975 ~ 1.026)	0.973
Sex	0.658 (0.229 ~ 1.894)	0.438
Hyperlipidemia	0.392 (0.060 ~ 2.564)	0.329
Stroke	0.161 (0.015 ~ 1.701)	0.129
Age	1.014 (0.958 ~ 1.073)	0.632
Anemia	0.113 (0.019 ~ 0.666)	0.016
Medications		
Aspirin	1.083 (0.316 ~ 3.719)	0.899
B-blocker	2.181 (0.734 ~ 6.479)	0.161
ACEI/ARB	1.871 (0.624 ~ 5.611)	0.264
CCB	1.255 (0.470 ~ 3.356)	0.650
Statins	0.258 (0.080 ~ 0.835)	0.024

ACEI: Angiotensin-converting enzyme inhibitors, ARB: Angiotensin II receptor antagonists, CCB: Calcium channel blocker.

## Discussion

The results of recent studies of the relationships between CKD and mortality and morbidity after ACS suggest that severe RD is an independent predictor of cardiovascular events [13-15]. RD is directly or indirectly involved in the development of hypertension, hyperlipidemia, endothelial disorders, and neuroendocrine disorders, which are all important contributors to the further advancement of RD in patients with ACS [4]. The American Kidney Foundation guidelines state that the prevalence of coronary heart disease increases with decreasing eGFR. A decrease in GFR to < 60 mL/min/1.73 m^2^ is an independent risk factor for coronary heart disease [16]. In this study, patients with more severe RD had higher mortality after ACS and were more likely to have hypertension, hyperlipidemia, ischemic heart disease, and cardiac dysfunction. Coronary angiography is relatively contraindicated in these patients. Surgeons should assess preoperative renal function and adequately communicate with patients and their families about the risks of angiography. It is important to ensure adequate hydration before and after coronary angiography, perform dialysis as needed, and prevent contrast-induced nephropathy and further damage to renal function. Patients with more severe RD undergo less frequent coronary angiography. Evaluation of renal function is an important aspect of risk assessment in patients with ACS. Current biomarkers of early changes in renal function include microalbuminuria and decreased eGFR [17].

Many patients become progressively malnourished as renal function decreases. The resulting decreases in albumin, prealbumin, and transferrin levels activate the inflammatory cascade [18,19]. Several aspects of progressive renal failure cause changes in plasma composition and endothelial structure and function that favor vascular injury [20-22]. However, the mechanisms underlying the impact of RD on the prognosis after ACS remain unclear. Significantly elevated serum levels of interleukin-6, CRP, and tumor necrosis factor-α have been observed in patients with renal failure, but no differences in prognosis have been reported between patients who underwent long-term dialysis and those who did not. In patients with CKD, the prevalences of vascular disease and malnutrition-inflammation-atherosclerosis syndrome increase as renal function decreases. Comorbidities may contribute to these changes [22,23]. Elevated inflammatory cytokine levels are associated with increased risk of cardiovascular disease [24,25]. The results of this study confirmed that severe RD is associated with high levels of inflammatory cytokines such as hs-CRP, but the cytokine levels were not independent predictors of MACE. Cys C is an independent predictor of early RD [26]. Our results also show higher Cys C levels in patients with more severe RD. Severe anemia and RD were independent risk factors for MACE, which is consistent with the results of previous studies [27,28].

The term “renal senescence” refers to a series of physiological and structural changes resulting in decreased renal function in aging individuals. Our findings are consistent with earlier reports that healthy individuals aged over 60 years have a GFR 20 to 30% lower than in individuals aged less than 50 years. The percentage of globally sclerosed glomeruli is frequently 10 to 40% in 60-year-old patients [29,30], which results in functional glomerulopenia. Sclerosis of the juxtamedullary glomeruli of aging patients results in a vascular connection between afferent and efferent arterioles that maintains an adequate rate of medullary blood flow while shunting blood past the obsolescent glomeruli. Elderly patients often have high morbidity associated with hypertension, diabetes mellitus, and other diseases, leading to the development of glomerular sclerosis. The elderly are also prone to RD due to stress or adverse drug effects, and such RD is difficult to reverse. Additionally, the prevalences of coronary heart disease and RD gradually increase with age. Evaluation of the risk factors and long-term prognosis of elderly patients with coronary heart disease and RD is therefore important. Our findings confirmed that in patients with ACS, more severe RD is associated with older age, hypertension, hyperlipidemia, and poor cardiac function. During follow-up, the incidence of MACE was significantly higher in patients with severe RD than in patients with mild RD. In patients with ACS, severe RD is an independent predictor of MACE and is associated with poor long-term prognosis. Evaluation of risk factors, overall renal function, and treatment options is therefore very important in patients with ACS.

This study has some limitations that should be discussed. First, the proportion of patients with unstable angina is higher in this study than that of previously reported studies. The American College of Cardiology/American Heart Association guidelines suggest that approximately 37% of patients with ACS have unstable angina [31]. We believe that the high proportion of patients with unstable angina may be attributed to the fact that the majority of patients were Beijing residents. It would therefore be useful for these patients to attend regular medical examinations, enabling earlier diagnosis and treatment. Second, 19 patients were lost to follow-up. Some of these patients lived in remote areas, with poor access to transport and communication media. Some patients may also have relocated during the relatively long follow-up period. Finally, patients with more severe RD underwent coronary angiography less frequently. This may be because RD is a relative contraindication to coronary angiography. Acute deterioration in renal function due to administration of contrast medium is a well-recognized complication after coronary angiography, particularly in patients with pre-existing CKD. We also considered these procedures in the multivariate Cox analysis.

## Conclusions

Among patients with ACS, severe RD was associated with advanced age, diabetes, hypertension, and cardiac dysfunction. Severe RD was an independent risk factor for MACE, and was associated with poor prognosis.

## Abbreviations

ACS: Acute coronary syndrome; RD: Renal dysfunction; eGFR: Estimated glomerular filtration rate; MACEs: Major adverse cardiac events; CKD: Chronic kidney disease; CVD: Cardiovascular disease; AKI: Acute kidney injury; SCr: Serum creatinine; NT-proBNP: N-terminal pro-B-type natriuretic peptide; hs-CRP: High-sensitivity C-reactive protein; Cys C: Cystatin C; ACC/AHA: The American College of Cardiology and the American Heart Association; STEMI: ST segment elevation myocardial infarction; NSTEMI: Non-ST Segment Elevation Myocardial Infarction; UA: Unstable angina; ACS: Acute coronary syndrome.

## Competing interests

All of authors declare that they have no competing interests.

## Authors’ contributions

Y. Liu, LG, and QX carried out the statistical analysis and drafted the manuscript. YF, JG, and XY collected the clinical data. MY, Y. Li, and YW participated in the design of the study and performed the statistical analysis. SW conceived of the study and participated in its design and coordination. All authors read and approved the final manuscript.

## Pre-publication history

The pre-publication history for this paper can be accessed here:

http://www.biomedcentral.com/1471-2369/15/78/prepub
